# Assessment of Dental Maturity of Children Aged 7-15 Years Using Demirjian Method in a Selected Iranian Population

**Published:** 2013-12

**Authors:** F Abesi, S Haghanifar, P Sajadi, A Valizadeh, S Khafri

**Affiliations:** a Dental Material Research Center, Dept. of Oral and Maxillofacial Radiology, Dental School, Babol University of Medical Sciences, Babol, Iran; b Academic member, Dept. of Social Medicine, Medical School, Babol University of Medical Sciences, Babol, Iran; c Dentist, Dental School, Babol University of Medical Sciences, Babol, Iran; d Dept. of Social Medicine, Medical School, Babol University of Medical Sciences, Babol, Iran

**Keywords:** Calcification, Age determination by teeth, Panoramic Radiography

## Abstract

**Statement of Problems:** Dental age can be estimated on the basis of the tooth mineralization level during the developmental process of the teeth. Among various radiological methods reported for the dental age determination in children, Demirjian method is widely used.

**Purpose:** To evaluate the applicability of Demirjian method in age estimation of the children aged 7-15 years in Babol, a northern city of Iran.

**Materials and Method:** A cross sectional study was performed on the panoramic radiographs of 168 individuals with 7-15 years old. Maturation of the seven permanent teeth on the left side of the mandible was determined according to the crown and root development stages; described by Demirjian method. The mean of the dental age (DA) according to the Demirjian was compared to the mean of chronological age (CA). Data were collected and analyzed using SPSS, V18. *P-values<*0.05 were considered significance.

**Results: **The mean and the SD of CA was 11.06±2.29 (boys: 11.08±2.31, girls: 11.03±2.28). The mean and the SD of DA was 11.44±2.85 (boys: 11.81±2.93, girls: 11.08±2.73) and the mean and the SD of DA minus CA for all of the children were 0.38±1.24 (boys: 0.72±1.2, girls: 0.05±1.21). Also, t-Test analysis showed the differences of the mean value of the estimated - chronological age difference was statistically significant between the boys and the girls group (*p*< 0.001).

**Conclusion:** Considering the determined differences between estimated dental age and chronological age in this study; Demirjian method can be applicable for estimation of dental age in girls and boys before their puberty in northern of Iran.

## Introduction

Chronological age (CA) is an important issue in most societies for school attendance, employment, social benefits and marriage [1]. However, there are many instances where CA might not be known due to un-documented or missing birth data. One of the most accurate, reliable and fast methods of age determination, especially in the growing children, is the dental method of age estimation [2]. 

On the other hand, the importance of dental age (DA) has been emphasized in forensic dentistry and pediatric dentistry and orthodontic treatments [3]. DA may be assessed either by tooth eruption time or by the progress of tooth calcification. Dental eruption is influenced by various factors such as crowding, extractions, ankylosis, ectopic positions, and persistence of primary teeth [4]. In addition, tooth eruption time cannot be realistic between the ages of 3 and 6, or after the age of 13. Therefore, tooth calcification is thought to be a more reliable criterion for determining DA [5].

In 1973, Demirjian introduced a dental age assessment method [6] based on the stages of tooth development in panoramic radiographs. This method has been widely used in different populations [3, 7-15].

Willems et al. [6] tested the validity of Demirjian’s method on Belgian Caucasian population and detected consistent overestimation of dental age in both genders. They offered new tables for each gender with age score directly expressed in years and they perceived higher accuracy with new adopted method. Mani et al. [7] performed Demirjian dental age estimation method on a sample of Malays individuals with 7 to 15years old age and reported that this method overestimated the age about 0.75 and 0.61 years among boys and girls, respectively. They suggested further modification of Demirjian method for Malays population.

Hegde et al. assessed the dental age of children from Belguam, India between 6 to 13years old by employing Demirjian method and conveyed a weak overestimation of 0.14 and 0.04 years for boys and girls, respectively, therefore recommended this method for dental age estimation in Belgaum children [11].

Bagherpour et al. [15] assessed dental age using Demirjian method among population in Mashhad, Iran and found that this method overestimated the age of boys by 0.34 years and girls by 0.25 years. The results indicated that Demirjian method is appropriate for estimating dental age of patients, especially those Bagherian et al. [16] evaluated dental age using Demirjian method among children in Rafsanjan, Iran and reported that this method overestimated the age by 0.15 and 0.21 years in boys and girls, respectively. They concluded Demirjian method as a clinically applicable method for the surveyed Iranian population.

The aim of this study was to assess the applicability of Demirjian method in Babol, a northern city of Iran, since little is known about this method’s applicability in this population.

## Materials and Method

This cross-sectional study was performed on 168 panoramic radiographs taken from 168 children (84 boys and 84 girls) referred to the Oral Maxillofacial Radiology clinic in Babol, Iran.

The protocol was reviewed and approved by the Medical Ethics Committee of Babol University of Medical Sciences. 

Inclusion criteria contained:

1. Age between 7 to 15 years old
2. No history of congenital and systemic disorders
3. To be the resident of Babol city
4. No missing left permanent mandibular teeth
5. Permission of the parents (written informed consent)

All of the radiographs were scored by one examiner using Demirjian method to obtain dental age.

Fifty subjects were re-examined after two weeks by the same examiner. Intra-examiner reproducibility was assessed using Cohen’s Kappa statistics for repeated maturity scores and was found to be 0.95.

In this method, seven left permanent mandibular teeth were scored between ‘’A’’ to “H” depending on the stage of calcification. Standards were given for each sex separately and sum of the scores of dental maturity was converted to dental age by using a conversion table. Scores used in this study were the revised scores reported by Demirjian and Goldstein [17].

Chronological ages of the children were obtained by subtracting birth dates from date that the radiograph were taken and were calculated into years. Children were also divided into 4 groups of 42 subjects according to their chronological ages (7-8.99, 9-10.99, 11-12.99, and 13-15).

All data were statistically analyzed by SPSS-18 software. The differences between chronological age and estimated dental ages were statistically compared using t-test and paired t-test. The p values less than 0.05 were considered to be statistically significant.

## Results

The mean and the SD of CA were 11.06±2.29 (boys: 11.08±2.31, girls: 11.03±2.28). The mean and the SD of DA were 11.44±2.85 (boys: 11.81±2.93, girls: 11.08± 2.73) and the mean and the SD of DA minus CA of children were 0.38±1.24 (boys: 0.72±1.2, girls: 0.05± 1.21).

Table 1 and 2 shows the comparison of dental ages using Demirjian method and chronological age in boys and girls, in each age groups respectively. 

**Table 1:** Comparison between chronological age and dental age in boys

**Age group**	**Chronological age** **Mean±SD**	**Dental age** **Mean±SD**	**Age difference** ^*^ **Mean±SD**	**CI 95% of age difference**	**p-value**
7-8.99	8.15±0.59	8.45±0.95	0.30±0.95	-0.12, 0.72	0.154
9-10.99	10.09±0.54	10.75±0.78	0.66±0.60	0.37, 0.94	0.001
11-12.99	12.12±0.54	12.40±1.41	0.27±1.30	-0.31, 0.87	0.344
13-15	14.07±0.64	15.73±1.43	1.66±1.29	1.07, 2.25	0.001

*Dental age minus chronological age; CI: confidence interval

In this study, Pearson correlation between dental age and chronological age was 0.90 (*p*< 0.001).There was a significant difference between the mean of chronological age and the dental age for girls and boys (*p*≤ 0.001). Also, t-test analysis showed that in comparison between the mean values of the differences between estimated ages and chronological ages, the difference between boys and girls (*p*< 0.001) was statistically significant.

## Discussion

General trend of the current findings revealed that Iranian children in Babol city indicated more advanced dental age compared to French-Canadian children as indicated by Demirjian [17]. This is in agreement with findings of other authors [3, 10-11, 15-16, 18) who indicated an overestimation of dental age ranging from 0.20 to 3.04 years in boys and 0.23 to 2.82 years in girls in their population [3, 8-20]. In contrast, underestimation of age was reported only in a Venezuelan population [21]. A possible explanation for the differences between Iranian and French-Canadian children might be attributed to the difference in ethnic group and/or effect of the considerable time gap between two studies on the dental development of these children. Furthermore, tooth development varies even between the different cities in the same country [17]. So that, in Iran there is a variety in ethnic groups and climate condition that can contribute to the difference in rate of overestimation. In the present study enrolled on a group of children living in Babol (a city located geographically in the northern part of Iran with a population around 200,000), rate of overestimation was 0.38±1.24. Whereas, in a study performed in Rafsanjan, Iran [16], rate of overestimation was 0.5±0.19 and in Isfahan, Iran [22] the value was 0.78±1.08. The comparison between the mean value of differences between estimated dental age and chronological age in this study showed statistically different values for boys versus girls (*p*< 0.001). Boys showed a difference of 0.72 years (262 days) and girls showed a minimal difference of 0.05 years (18 days).

Table 1 show that the rate of overestimation was less in the up-to-13 year age groups and was clinically acceptable, however, for the age range of 13-15 years; more researches are needed.

For girls, Demirjian method was an applicable method in dental age estimation, whilst higher accuracy was observed for the individuals younger than 11 years old (Table 2).

In general, the accuracy of Demirjian method was decreased in estimating the dental age in girls over 11 and boys over 13 years old. The fact that overestimation was more pronounced in grown-up children could perhaps be linked to the puberty and this result was in agreement with the studies Bagherian et al. [16]. According to that study puberty could influence the dental age estimation but they reported that the overestimation was more common in younger children.

The present study was consistent with the studies enrolled by Nikanen et al. [23] and Jayaraman et al. [19].

Nikanen et al. [23] explained that the self-weighted scores of Demirjian dataset were based on the midpoint between two successive stages and the proper score was allocated to the higher development stage. In addition, the period between each individual stage rises with the age of the subject and so this phenomenon could end to an overestimation of age expected for adult children.

**Table 2:** Comparison between chronological age and dental age in girls

**Age group**	**Chronological age** **Mean±SD**	**Dental age** **Mean±SD**	**Age difference** **Mean±SD**	**95% CI of age difference**	**p.value**
7-8.99	08.11±0.98	8.14±0.98	0.03±0.74	-0.30, 0.37	0.840
9-10.99	10±0.52	9.90±0.87	-0.09±0.94	-0.52, 0.33	0.648
11-12.99	11.95±0.58	11.50±1.11	-0.45±1.30	-1.06, 0.16	0.140
13-15	13.97±0.56	14.65±1.78	0.68±1.47	0.03, 1.34	0.041

It must be noted that other factors such as socio-economic status, nutrition, dietary habits and life style could result in these differences as well. Similarly, sample size and number of subjects in each group as a consequence, may modify the overall result of the study. Most studies, purposed to test the applicability of a dataset, had variable number of subjects in each age group [10-12]. Hence, in the current study, we standardized the procedure with an identical number of DPTs in each age group so that the appropriate overall differences between chronological age (CA) and dental age (DA) could be established. Moreover, it is necessary to mention that the current study is the only research in which each group has the same total number of girls and boys.

In conclusion, according to the differences between the estimated dental age and the chronological age in this study, Demirjian method can be applicable for dental age estimation in girls and boys before their puberty in northern of Iran.

## References

[1] Willems G (2001). A review of the most commonly used dental age estimation techniques. J Forensic Odontostomatol.

[2] Nik-Hussein NN, Kee KM, Gan P (30). Validity of Demirjian and Willems methods for dental age estimation for Malaysian children aged 5-15 years old. Forensic Sci Int.

[3] Koshy S, Tandon S (1998). Dental age assessment: the applicability of Demirjian's method in south Indian children. Forensic Sci Int.

[4] Uysal T, Yagci A, Ramoglu SI (2009). Dental maturation in pa-tients with unilateral posterior crossbite. World J Orthod.

[5] Nur B, Kusgoz A, Bayram M, Celikoglu M, Nur M, Kayipmaz S (2012). Validity of demirjian and nolla methods for dental age estimation for Northeastern Turkish children aged 5-16 years old. Med Oral Patol Oral Cir Bucal.

[6] Willems G, Van Olmen A, Spiessens B, Carels C (2001). Dental age estimation in Belgian children: Demirjian's technique revisited. J Forensic Sci.

[7] Mani SA, Naing L, John J, Samsudin AR (2008). Comparison of two methods of dental age estimation in 7-15-year-old Malays. Int J Paediatr Dent.

[8] Eid RM, Simi R, Friggi MN, Fisberg M (2002). Assessment of dental maturity of Brazilian children aged 6 to 14 years using Demirjian's method. Int J Paediatr Dent.

[9] Davis PJ, Hägg U (1994). The accuracy and precision of the "Demirjian system" when used for age determination in Chinese children. Swed Dent J.

[10] Hägg U, Matsson L (1985). Dental maturity as an indicator of chronological age: the accuracy and precision of three methods. Eur J Orthod.

[11] Hegde RJ, Sood PB (2002). Dental maturity as an indicator of chronological age: radiographic evaluation of dental age in 6 to 13 years children of Belgaum using Demirjian methods. J Indian Soc Pedod Prev Dent.

[12] Leurs IH, Wattel E, Aartman IH, Etty E, Prahl-Andersen B (2005). Dental age in Dutch children. Eur J Orthod.

[13] Liversidge HM, Speechly T, Hector MP (1999). Dental maturation in British children: are Demirjian's standards applicable?. Int J Paediatr Dent.

[14] Prabhakar AR, Panda AK, Raju OS (2002). Applicability of Demirjian's method of age assessment in children of Davangere. J Indian Soc Pedod Prev Dent.

[15] Bagherpour A, Imanimoghaddam M, Bagherpour MR, Einolghozati M (2010). Dental age assessment among Iranian children aged 6-13 years using the Demirjian method. Forensic Sci Int.

[16] Bagherian A, Sadeghi M (5). Assessment of dental maturity of children aged 3.5 to 13.5 years using the Demirjian method in an Iranian population. J Oral Sci.

[17] Demirjian A, Goldstein H (1976). New systems for dental maturity based on seven and four teeth. Ann Hum Biol.

[18] Nyström M, Ranta R, Kataja M, Silvola H (1988). Comparisons of dental maturity between the rural community of Kuhmo in northeastern Finland and the city of Helsinki. Community Dent Oral Epidemiol.

[19] Jayaraman J, King NM, Roberts GJ, Wong HM (2011). Dental age assessment: are Demirjian's standards appropriate for southern Chinese children?. J Forensic Odontostomatol.

[20] Maber M, Liversidge HM, Hector MP (2006). Accuracy of age estimation of radiographic methods using developing teeth. Forensic Sci Int.

[21] Cruz-Landeira A, Linares-Argote J, Martínez-Rodríguez M, Rodríguez-Calvo MS, Otero XL, Concheiro L (2010). Dental age estimation in Spanish and Venezuelan children Comparison of Demirjian and Chaillet's scores. Int J Legal Med.

[22] Javadinejad S, Karami M, Hashemnia N (2010). Association between body mass index and dental development in 7-15 year old children in the city of Isfahan-Iran in the year 2008. J Mashhad Dent Sch.

[23] Nykänen R, Espeland L, Kvaal SI, Krogstad O (1998). Validity of the Demirjian method for dental age estimation when applied to Norwegian children. Acta Odontol Scand.

